# Supplementary material for: Rapid in-air ultrasound holography measurement and camera-in-the-loop generation using thermography
Source: Commun Eng. 2025 Jun 5;4:101. doi: 10.1038/s44172-025-00439-w (PMC12141450; doi:10.1038/s44172-025-00439-w)
Supplement: Supplementary file 2 — Supplementary Information [file 44172_2025_439_MOESM2_ESM.pdf]

Supplementary Material: Rapid in-air Ultrasound Holography Measurement and Camera-in-the-loop Generation using Thermography

Zak Morgan[1*], Youngjun Cho[1] and Sriram Subramanian[1]

[1*]Department of Computer Science, University College London, Gower St, London, WC1E 6BT, London, United Kingdom.

*Corresponding author(s). E-mail(s): zak.morgan.17@ucl.ac.uk;
Contributing authors: youngjun.cho@ucl.ac.uk; s.subramanian@ucl.ac.uk;

Supplementary Table 1

	1kPa Target		6kPa Target	
Mesh Hole Size	Pressure (Pa)	Transmission (%)	Pressure (Pa)	Transmission (%)
empty	975	100	4375	100
75μm	950	97.4	4279	97.8
50μm	915	93.8	4189	95.8
25μm	544	55.8	1913	43.7
5μm	259	26.6	1035	23.7
2.5μm	140	14.4	562	12.8

Table 1: **Pressure measured with a microphone of a focal point after passing through a mesh with various hole sizes.** Meshes with 75, 50, 25, 5 and 2.5μm holes were chosen. [1] used $77\mu m$ and so the 75 mesh here allows for comparison. A suitably sized impedance tube does not exist in order to measure the reflection and absorption co-efficient of a material for 40khz ultrasound. Thus an approximation was made by measuring the intensity of a focal point after passing through a given mesh both with a microphone. The results show as expected a decreasing amount of transmission as hole sizes in the mesh get smaller.

Supplementary Figure 1

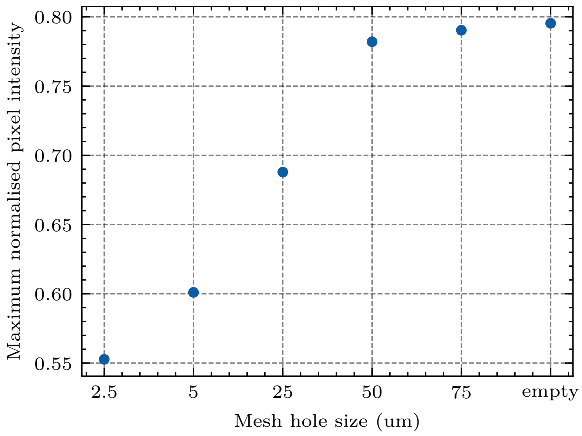

Fig. 1: **Maximum normalised schlieren pixel intensity of a focal point passing through a mesh against mesh hole size.** This data confirms that as mesh hole size decreases, transmission decreases, and also that 75um and 50um meshes do not inhibit the mesh too much as predicted by the analytical acoustic model.

Supplementary Figure 2

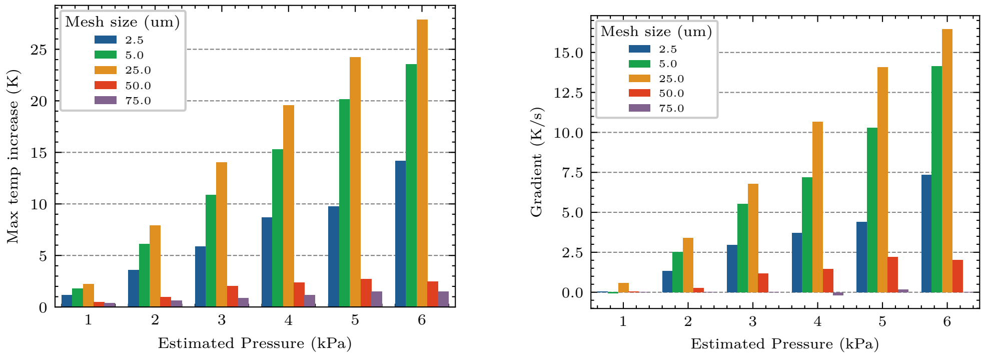

(a) (b)

Fig. 2: **Maximum relative temperature increase and gradient in a mesh against focal point pressure.** An ultrasonic focal point with an estimated magnitude of 1-6kPa in 1kPa increments was created 15cm away from the centre of a PAT board using GS-PAT. The mesh was then inserted to intersect this focal point. a.) The gradient of the initial temperature rise was calculated after 10 frames. The results show that the 25μm mesh is the only mesh sensitive enough for low pressure measurements via temperature gradient b.) The steady state temperature reached after approximately 10 seconds was also recorded shows that only the 25, 5 and 2.5 μm mesh are suitable, with the 25μm being optimal.

Supplementary Notes 1

First we look the simple situation of one medium (air) where temperature rise ($\frac{\partial T_1}{\partial t}$) in air is linked to the root mean square (RMS) pressure in air ($P_{1,\text{RMS}}$):

$$p_{1,\text{RMS}} = \sqrt{\frac{\rho_1^2 c_1 C_1 V_1}{\alpha_1(0)(1-\mathrm{e}^{-2a_1 D_1})A_1}}\sqrt{\frac{\partial T_1}{\partial t}} \tag{1}$$

As in [2] here the sub-script refers to material and so P_1 would be pressure in air, and P_2 would be the pressure in the nylon mesh. Here ρ is the material density, c is the speed of sound, α is the attenuation coefficient and paired with D the thickness of the mesh equates to the attenuation suffered whilst travelling through the mesh, $\alpha(0)$ is the absorbtion coefficient at 0 degrees, V and A are the volume and surface area respectively and C is the specific heat capacity.

Whilst in [1] there is an assumption that the temperature rise in air is primarily responsible for the temperature increase in the mesh (when $\theta = 0$), here we instead model as the heat being generated in the mesh itself. Thus the equation for the pressure in the mesh is:

$$p_{2,\mathrm{RMS}} = \sqrt{\frac{\rho_2^2 c_2 C_2 V_2}{\alpha_2(0)(1 - e^{-2a_2 D_2}) A_2}} \sqrt{\frac{\partial T_2}{\partial t}} \tag{2}$$

Since our ground truth measurements from the microphone measure P_1, and our thermal camera measures $\frac{\partial T_2}{\partial t}$, we must move convert from P_2 to P_1. The intensity of the wave in both materials must be the same thus giving:

$$\frac{P_{1,\mathrm{RMS}}^2}{\rho_1 c_1} = \frac{P_{2,\mathrm{RMS}}^2}{\rho_2 c_2} \tag{3}$$

Which can be re-arrange for P_1 to give:

$$P_{1,\mathrm{RMS}} = \sqrt{\frac{P_{2,\mathrm{RMS}}^2 \rho_1 c_1}{\rho_2 c_2}} \tag{4}$$

Substituting Eq 4 into Eq 1 and re-arranging gives a final equation:

$$p_{1,\mathrm{RMS}} = \sqrt{\frac{C_2 \rho_2 V_2 \rho_1 c_1}{\alpha_2(0)(1 - e^{-2a_2 D_1}) A_1}} \sqrt{\frac{\partial T_2}{\partial t}} \tag{5}$$

Supplementary Figure 3

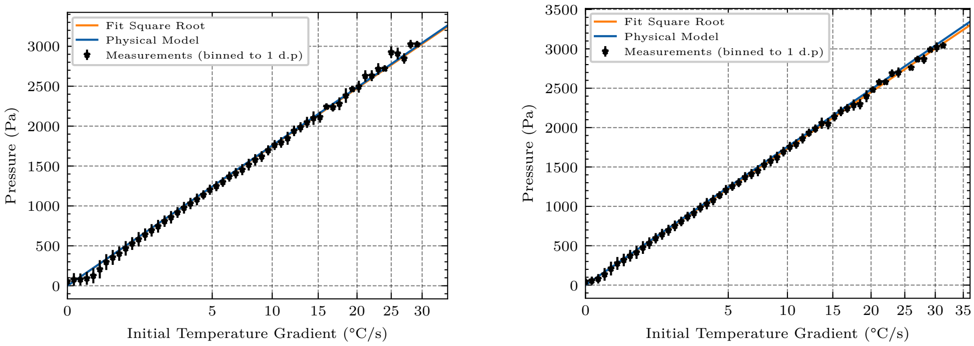

(a) = (b) =

Fig. 3: **Temperature gradient against pressure with and without non-uniform time sampling.** a.) The initial temperature gradient without non-uniform time sampling demonstrates a non-linear relationship in the low pressure values, and additionally instability at higher pressures. b) Using non-uniform time sampling demonstrates the ability to compensate for noise at low temperatures, and compensating for numerical differentiation errors at large values, resulting in a distribution that fits much closer to a linear line of best fit.

Supplementary Figure 4

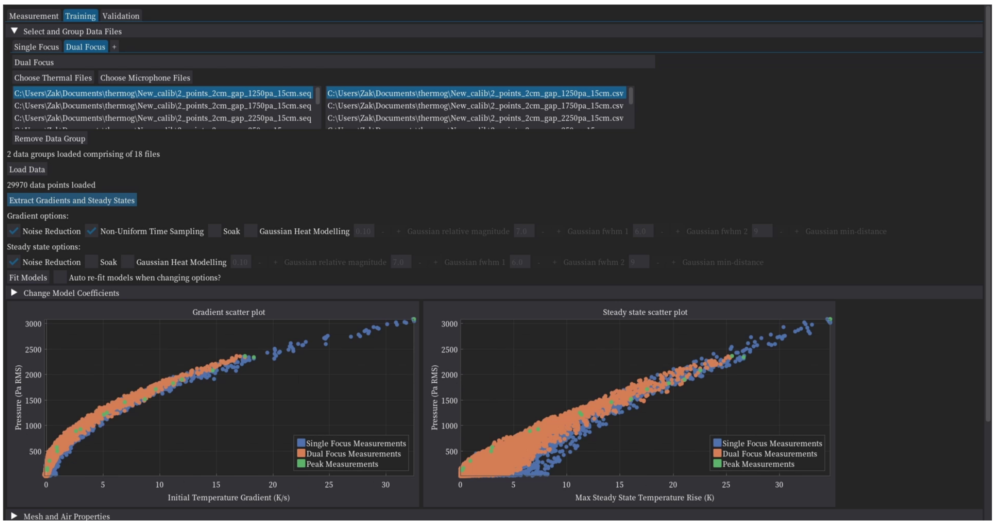

(a)

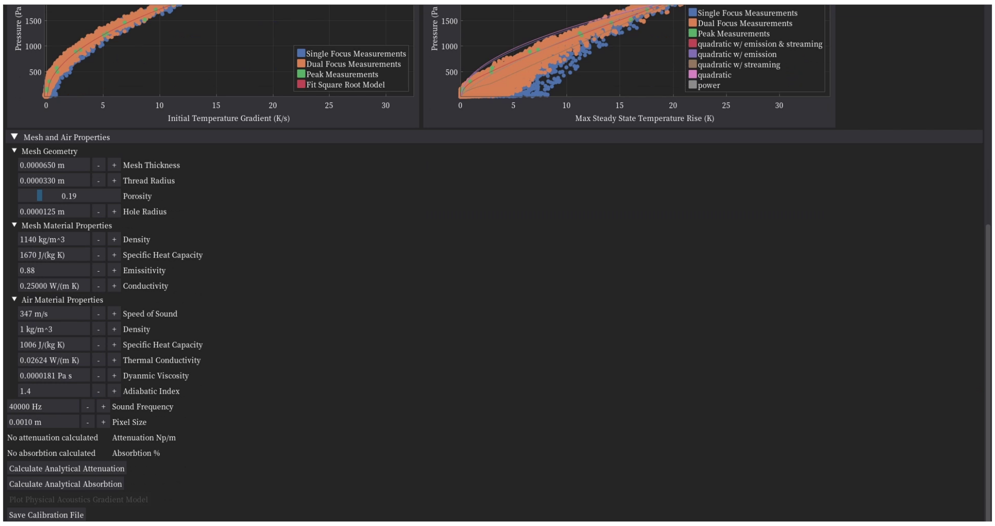

(b)

Fig. 4: **GUI of fitting screen for thermal pressure measurement software.** a.) View for fitting coefficients. b.) View for selecting mesh properties for the physical model.

Supplementary Figure 5

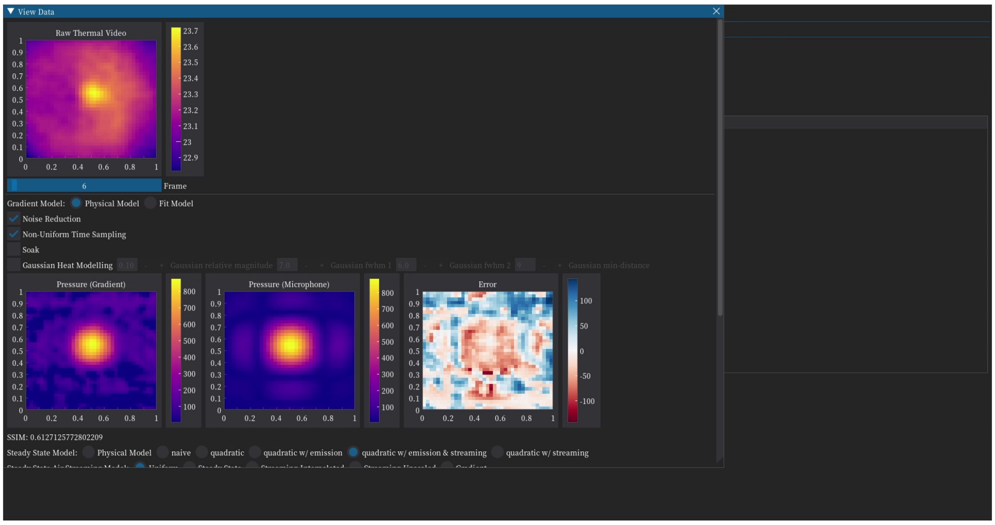

(a)

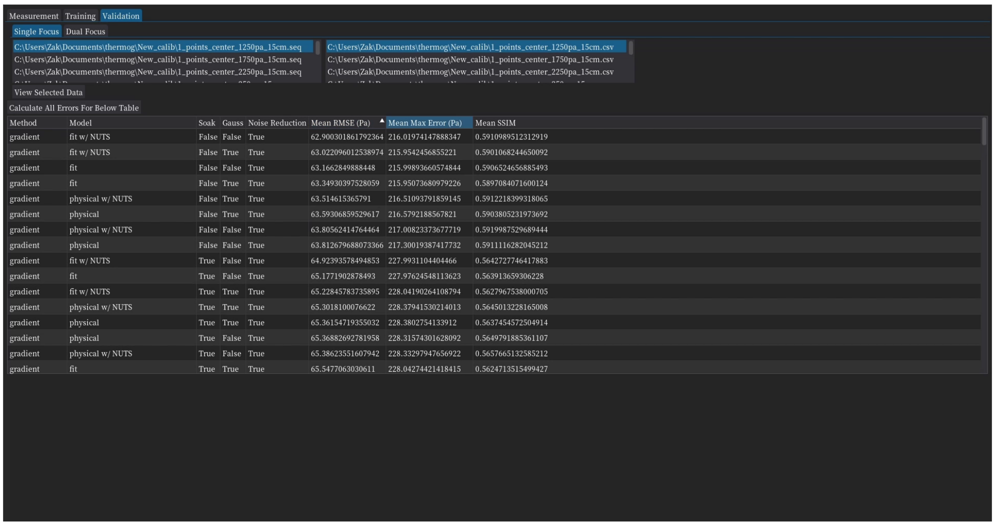

Method	Model	Soak	Gauss	Noise Reduction	Mean RMSE (Pa)	Mean Max Error (Pa)	Mean SSIM
gradient	fit w/ NUTS	False	False	True	62.900301861792364	216.01974147888347	0.5910989512312919
gradient	fit w/ NUTS	False	True	True	63.022096012538974	215.9542456855221	0.5901068244650092
gradient	fit	False	False	True	63.1662849888448	215.99893660574844	0.5906524656885493
gradient	fit	False	True	True	63.34930397528059	215.95073680979226	0.5897084071600124
gradient	physical w/ NUTS	False	True	True	63.514615365791	216.51093791859145	0.5912218399318065
gradient	physical	False	True	True	63.59306859529617	216.5792188567821	0.5903805231973692
gradient	physical w/ NUTS	False	False	True	63.80562414764464	217.00823373677719	0.5919987529689444
gradient	physical	False	False	True	63.812679688073366	217.30019387417732	0.5911116282045212
gradient	fit w/ NUTS	True	False	True	64.92393578494853	227.9931104404466	0.5642727746417883
gradient	fit	True	False	True	65.1771902878493	227.97624548113623	0.563913659306228
gradient	fit w/ NUTS	True	True	True	65.22845783735895	228.04190264108794	0.5627967538000705
gradient	physical w/ NUTS	True	True	True	65.3018100076622	228.37941530214013	0.5645013228165008
gradient	physical	True	True	True	65.36154719355032	228.3802754133912	0.5637454572504914
gradient	physical	True	False	True	65.36882692781958	228.31574301628092	0.5649791885361107
gradient	physical w/ NUTS	True	False	True	65.38623551607942	228.33297947656922	0.5657665132585212
gradient	fit	True	True	True	65.5477063030611	228.04274421418415	0.5624713515499427

(b)

Fig. 5: **GUI of validation screen for thermal pressure measurement software.** a.) View for validating fits and models showing the ground-truth, the estimated and the error. b.) Full ablation table generated for all attributes.

Supplementary Figure 6

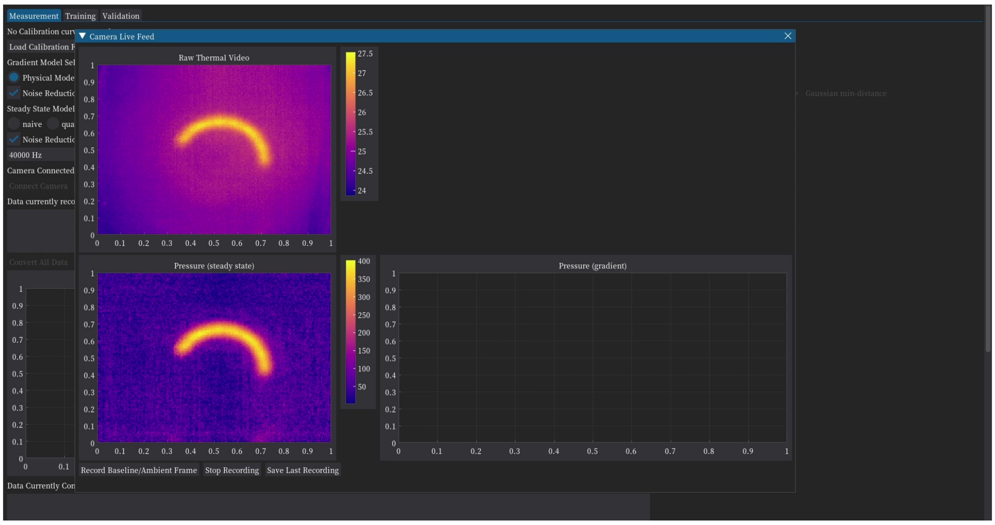

(a)

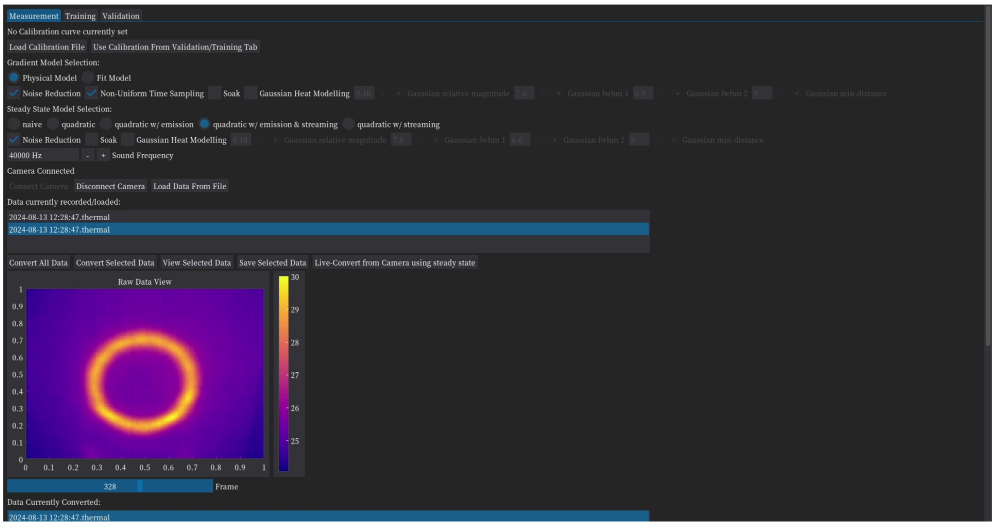

(b)

Fig. 6: **GUI of live-data acquisition for thermal pressure measurement software.** a.) View for live recording from a thermal camera. b.) Viewing and converting saved recorded data.

Supplementary Figure 7

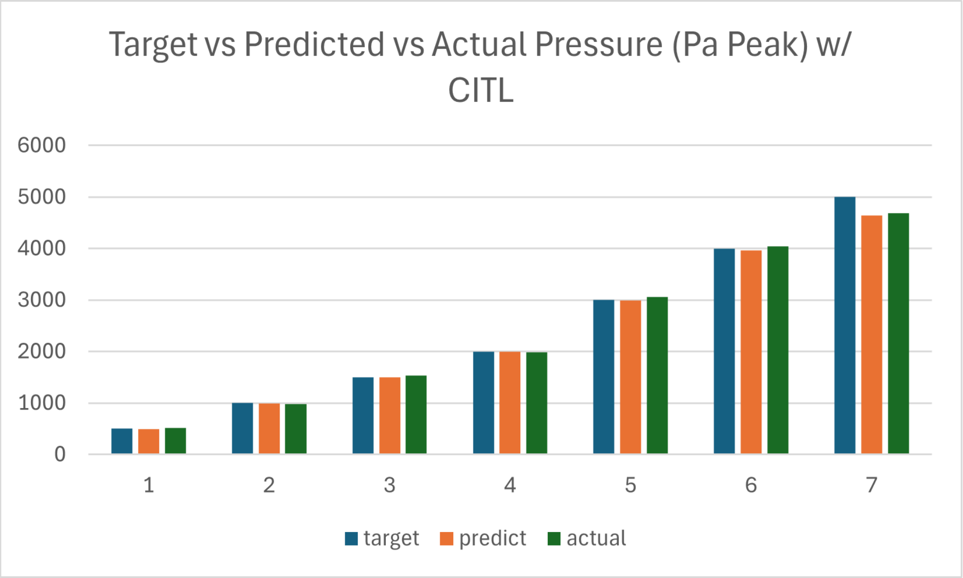

Fig. 7: **Target, predicted and actual pressure for camera in the loop single point holography wrapping GS_PAT.**

Supplementary Table 2

Method	Model	Soak	Gauss	Noise Reduction	Mean RMSE (Pa)	Mean Max Error (Pa)	Mean FWHM error (mm)	Mean SSIM
Gradient	NUTS	False	False	True	64	227	0.37	0.59
Gradient	Raw	False	False	True	64	228	0.42	0.59
Gradient	NUTS	True	False	True	66	235	0.38	0.56
Gradient	Raw	True	False	True	66	235	0.42	0.56
Gradient	Conduction	False	False	True	67	232	0.41	0.59
Gradient	Conduction	True	False	True	67	241	0.42	0.58
Gradient	Conduction & NUTS	False	False	True	67	232	0.38	0.59
Gradient	Conduction & NUTS	True	False	True	68	241	0.39	0.58
Steady State	[2] w/ emission, convection	True	True	True	79	257	0.69	0.60
Steady State (PM)	gradient	True	True	True	79	276	0.41	0.56
Gradient	NUTS	True	False	False	80	290	0.53	0.49
Gradient	Raw	True	False	False	80	290	0.55	0.49
Steady State (PM)	steady state	True	True	True	81	276	0.43	0.56
Steady State	[2] w/ convection	False	True	True	81	276	1.28	0.60
Steady State (PM)	[3] image	True	True	True	81	282	0.41	0.56
Steady State	[2] w/ convection	True	True	False	82	278	1.35	0.59
Steady State	[2] w/ convection	True	True	True	82	278	1.33	0.59
Steady State (PM)	[3]	True	True	True	82	283	0.43	0.56
Steady State	[2] w/ emission, convection	True	True	False	83	266	0.72	0.59
Gradient	Conduction	True	False	False	83	314	0.56	0.51
Gradient	Conduction & NUTS	True	False	False	84	314	0.55	0.51
Steady State	[2] w/ convection	False	True	False	84	278	1.30	0.59
Steady State	[2] w/ emission, convection	False	True	True	86	267	0.65	0.59
Steady State (PM)	gradient	False	True	True	86	276	0.44	0.57
Steady State (PM)	steady state	False	True	True	87	277	0.43	0.57
Steady State (PM)	gradient	True	True	False	87	292	0.39	0.53
Steady State (PM)	steady state	True	True	False	88	293	0.41	0.53
Steady State (PM)	[3] image	False	True	True	89	286	0.43	0.57
Gradient	NUTS	False	False	False	89	284	0.47	0.49
Gradient	Raw	False	False	False	89	284	0.51	0.49
Steady State (PM)	[3] image	True	True	False	89	299	0.40	0.53
Steady State	[2] w/ convection	True	False	True	89	311	0.98	0.58
Steady State	[2] w/ convection	True	False	False	90	311	1.01	0.57
Steady State (PM)	[3]	True	True	False	90	300	0.40	0.53
Steady State (PM)	[3]	False	True	True	90	287	0.46	0.57
Steady State	[2] w/ convection	False	False	True	91	312	0.93	0.57
Steady State	[2] w/ convection	False	False	False	94	312	0.97	0.55
Gradient	Conduction	False	False	False	94	313	0.54	0.50
Steady State (PM)	uniform	True	True	True	94	306	0.83	0.54
Gradient	Conduction & NUTS	False	False	False	94	312	0.51	0.50
Steady State	[2] w/ emission, convection	False	True	False	95	278	0.72	0.57
Steady State	[2] w/ emission, convection	True	False	True	95	300	0.55	0.56
Steady State (PM)	gradient	False	True	False	98	300	0.46	0.53
Steady State (PM)	steady state	False	True	False	100	303	0.48	0.53
Steady State	[2] w/ emission, convection	True	False	False	100	309	0.55	0.55
Steady State (PM)	[3] image	False	True	False	101	312	0.47	0.53
Steady State (PM)	uniform	True	True	False	102	324	0.77	0.52
Steady State (PM)	uniform	False	True	True	103	309	0.89	0.55
Steady State (PM)	[3]	False	True	False	103	313	0.47	0.53
Steady State	[2] w/ emission, convection	False	False	True	105	311	0.57	0.55
Steady State (PM)	gradient	True	False	True	113	340	0.96	0.57
Steady State	[2] w/ emission, convection	False	False	False	114	322	0.62	0.52
Steady State (PM)	uniform	False	True	False	115	331	0.86	0.52
Steady State (PM)	steady state	True	False	True	115	353	0.89	0.56
Steady State (PM)	[3] image	True	False	True	121	358	1.11	0.55
Steady State (PM)	[3]	True	False	True	124	362	1.12	0.55
Steady State (PM)	steady state	True	False	False	126	381	0.83	0.50
Steady State (PM)	gradient	False	False	True	127	354	1.06	0.54
Steady State (PM)	steady state	False	False	True	131	368	0.98	0.53
Steady State (PM)	[3] image	True	False	False	131	385	1.02	0.50
Steady State (PM)	[3]	True	False	False	135	389	0.99	0.49
Steady State (PM)	[3] image	False	False	True	135	371	1.23	0.52
Steady State (PM)	gradient	True	False	False	137	552	0.86	0.53
Steady State (PM)	uniform	True	False	True	138	378	2.18	0.52
Steady State (PM)	[3]	False	False	True	139	376	1.23	0.52
Steady State (PM)	gradient	False	False	False	141	380	1.00	0.48
Steady State (PM)	steady state	False	False	False	144	396	0.96	0.47
Steady State (PM)	uniform	True	False	False	148	404	2.01	0.47
Steady State (PM)	[3] image	False	False	False	149	399	1.17	0.47
Steady State (PM)	[3]	False	False	False	153	403	1.14	0.47
Steady State (PM)	uniform	False	False	True	154	392	2.33	0.49
Steady State (PM)	uniform	False	False	False	167	418	2.18	0.44
Steady State	[2] w/ emission	True	True	True	220	532	2.37	0.47
Steady State	[2] w/ emission	True	True	False	233	548	2.32	0.45
Steady State	[2]	True	True	True	244	578	2.76	0.45
Steady State	[2] w/ emission	False	True	True	246	548	2.51	0.45
Steady State	[2] w/ emission	True	False	True	256	574	3.31	0.42
Steady State	[2]	True	True	False	258	595	2.72	0.43
Steady State	[2] w/ emission	False	True	False	268	570	2.49	0.43
Steady State	[2] w/ emission	True	False	False	269	594	3.26	0.40
Steady State	[2]	False	True	True	271	594	2.91	0.43
Steady State	[2] w/ emission	False	False	True	282	590	3.50	0.40
Steady State	[2]	True	False	True	282	618	3.73	0.40
Steady State	[2]	False	True	False	294	616	2.91	0.41
Steady State	[2]	True	False	False	296	638	3.70	0.39
Steady State	[2] w/ emission	False	False	False	303	615	3.49	0.39
Steady State	[2]	False	False	True	309	634	3.95	0.39
Steady State	[2]	False	False	False	332	660	3.95	0.37

Table 2: **Ablation table of all thermal to pressure methods and parameters and their errors ordered by Mean RMSE.** Model determines the sampling method for the gradient technique, the types of cooling considered for the steady state, and the convection coefficient distribution used for the physical model (further information in the supplementary information). "Soak" determines if compensating for the general temperature increase in the material, "Gauss" if a gaussian model was used to estimate heat diffusion and "Noise Reduction" if noise reduction was used

References

[1] Onishi, R. *et al.* Two-dimensional measurement of airborne ultrasound field using thermal images. *Phys. Rev. Appl.* **18**, 044047 (2022). URL https://link.aps.org/doi/10.1103/PhysRevApplied.18.044047.

[2] Melde, K., Qiu, T. & Fischer, P. Fast spatial scanning of 3D ultrasound fields via thermography. *Applied Physics Letters* **113**, 133503 (2018). URL https://doi.org/10.1063/1.5046834.

[3] Pittera, D., Georgiou, O., Abdouni, A. & Frier, W. "i can feel it coming in the hairs tonight": Characterising mid-air haptics on the hairy parts of the skin. *IEEE Transactions on Haptics* **15**, 188–199 (2022).
