# Supplementary material for: Rapid in-air ultrasound holography measurement and camera-in-the-loop generation using thermography
Source: Commun Eng. 2025 Jun 5;4:101. doi: 10.1038/s44172-025-00439-w (PMC12141450; doi:10.1038/s44172-025-00439-w)
Supplement: Supplementary file 3 — Description of Additional Supplementary Files [file 44172_2025_439_MOESM3_ESM.pdf]

Description of Additional Supplementary Files

File name: Supplementary Movie 1

Description: A video abstract showcasing the work

File name: Supplementary Movie 2

Description: A video showcasing how to use the GUI of the software which implements the techniques developed in the work.
